# Supplementary material for: Phase Angle, Handgrip Strength, and Other Indicators of Nutritional Status in Cancer Patients Undergoing Different Nutritional Strategies: A Systematic Review and Meta-Analysis
Source: Nutrients. 2023 Apr 6;15(7):1790. doi: 10.3390/nu15071790 (PMC10097099; doi:10.3390/nu15071790)
Supplement: Supplementary file 1 [file nutrients-15-01790-s001.zip › Table S1.pdf]

Table S1. Excluded studies and reasons for exclusion.

Caccialanza, R., Cereda, E., Caraccia, M., Klersy, C., Nardi, M., Cappello, S., Borioli, V., Turri, A., Imarisio, I., Lasagna, A., Saddi, J., Arcaini, L., Benazzo, M., Stragliotto, S., Zagonel, V., & Pedrazzoli, P. (2019). Early 7-day supplemental parenteral nutrition improves body composition and muscle strength in hypophagic cancer patients at nutritional risk. Supportive Care in Cancer: Official Journal of the Multinational Association of Supportive Care in Cancer, 27(7), 2497–2506. https://doi.org/10.1007/s00520-018-4527-0.
Reason for exclusion: No control group.
Capello, S., Cereda, E., Colombo, S., Klersy, C., & Imarisio, I. (n.d.). Counseling with or without systematic use of oral supplements in head-neck cancer patients undergoing radiotherapy. Retrieved March 18, 2023, from https://www.proquest.com/docview/2061436196
Reason for exclusion: Abstract.
Cornejo-Pareja, I., Ramirez, M., Camprubi-Robles, M., Rueda, R., Vegas-Aguilar, I. M., & Garcia-Almeida, J. M. (2021). Effect on an Oral Nutritional Supplement with β-Hydroxy-β-methylbutyrate and Vitamin D on Morphofunctional Aspects, Body Composition, and Phase Angle in Malnourished Patients. Nutrients, 13(12), 4355. https://doi.org/10.3390/nu13124355
Reason for exclusion: No cancer patients.
Della Valle, S., Colatruglio, S., La Vela, V., Tagliabue, E., Mariani, L., & Gavazzi, C. (2018). Nutritional intervention in head and neck cancer patients during chemo-radiotherapy. NUTRITION, 51–52, 95–97. https://doi.org/10.1016/j.nut.2017.12.012.
Reason for exclusion: No control group.
Di Renzo, L., Marchetti, M., Cioccoloni, G., Gratteri, S., Capria, G., Romano, L., Soldati, L., Mele, M. C., Merra, G., Cintoni, M., & De Lorenzo, A. (2019). Role of phase angle in the evaluation of effect of an immuno-enhanced formula in post-surgical cancer patients: A randomized clinical trial. EUROPEAN REVIEW FOR MEDICAL AND PHARMACOLOGICAL SCIENCES, 23(3), 1322–1334.
Reason for exclusion: No control group.
Finger, A. (2021). 917P - Head and neck cancer patients under (chemo-)radiotherapy undergoing nutritional intervention: Results from the prospective randomized HEADNUT-trial.
Reason for exclusion: Poster
Kutz, L. M., Abel, J., Schweizer, D., Tribius, S., Krüll, A., Petersen, C., & Löser, A. (2022). Quality of life, HPV-status and phase angle predict survival in head and neck cancer patients under (chemo)radiotherapy undergoing nutritional intervention: Results from the prospective randomized HEADNUT-trial. Radiotherapy and Oncology: Journal of the European Society for Therapeutic Radiology and Oncology, 166, 145–153. https://doi.org/10.1016/j.radonc.2021.11.011
Reason for exclusion: Not valid data
Löser, A., Abel, J., Kutz, L. M., Krause, L., Finger, A., Greinert, F., Sommer, M., Lorenz, T., Culmann, E., von Grundherr, J., & al., et. (2021). Head and neck cancer patients under (chemo-) radiotherapy undergoing nutritional intervention: Results from the prospective randomized HEADNUT-trial. Radiotherapy and Oncology, 159 CC-, 82-90. https://doi.org/10.1016/j.radonc.2021.03.019

Reason for exclusion: No valid data.
Orell, H., Schwab, U., Saarilahti, K., Österlund, P., Ravasco, P., & Mäkitie, A. (2019). Nutritional Counseling for Head and Neck Cancer Patients Undergoing (Chemo) Radiotherapy-A Prospective Randomized Trial. Frontiers in Nutrition, 6, 22. https://doi.org/10.3389/fnut.2019.00022
Reason for exclusion: No control group.
Tumas, J., Tumiene, B., Jurkeviciene, J., Jasiunas, E., & Sileikis, A. (2020). Nutritional and immune impairments and their effects on outcomes in early pancreatic cancer patients undergoing pancreatoduodenectomy. Clinical Nutrition, 39(11), 3385–3394. https://doi.org/10.1016/j.clnu.2020.02.029
Reason for exclusion: Not valid data.
