# Supplementary figures and images for: Phase Angle, Handgrip Strength, and Other Indicators of Nutritional Status in Cancer Patients Undergoing Different Nutritional Strategies: A Systematic Review and Meta-Analysis
Source: Nutrients. 2023 Apr 6;15(7):1790. doi: 10.3390/nu15071790 (PMC10097099; doi:10.3390/nu15071790)

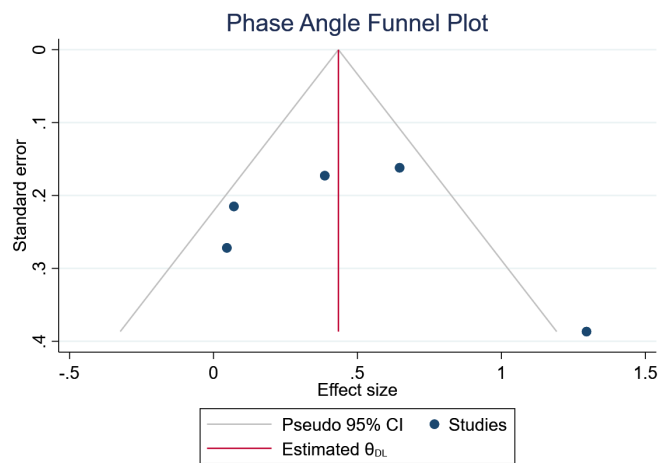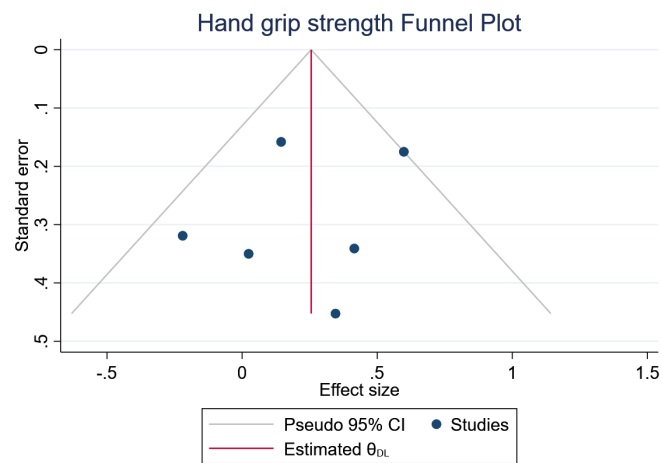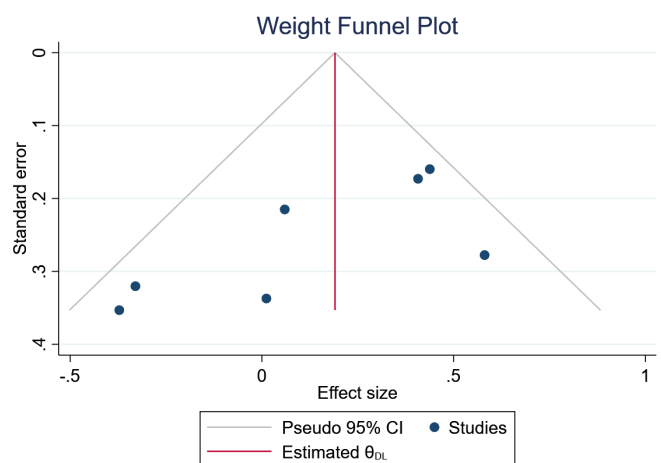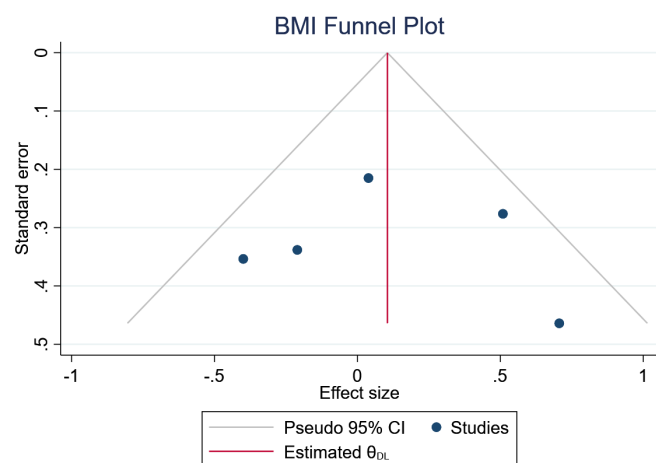

Figure S1: Funnel plot for phase angle, hand grip strength, weight and BMI.

Supplement: Supplementary file 1 [file nutrients-15-01790-s001.zip › Figure S1.pdf]
